# The Tetracycline Resistance Gene, *tet*(W) in *Bifidobacterium animalis* subsp. *lactis* Follows Phylogeny and Differs From *tet*(W) in Other Species

**DOI:** 10.3389/fmicb.2021.658943

**Published:** 2021-07-15

**Authors:** Katrine Nøhr-Meldgaard, Carsten Struve, Hanne Ingmer, Yvonne Agersø

**Affiliations:** ^1^Chr. Hansen A/S, Hørsholm, Denmark; ^2^Department of Veterinary and Animal Sciences, University of Copenhagen, Frederiksberg, Denmark

**Keywords:** antimicrobial, antibiotic, resistance evolution, non-pathogenic bacteria, ribosomal protection, intrinsic resistance

## Abstract

The tetracycline resistance gene *tet*(W) encodes a ribosomal protection protein that confers a low level of tetracycline resistance in the probiotic bacterium *Bifidobacterium animalis* subsp. *lactis.* With the aim of assessing its phylogenetic origin and potential mobility, we have performed phylogenetic and *in silico* genome analysis of *tet*(W) and its flanking genes. *tet*(W) was found in 41 out of 44 examined *B. animalis* subsp. *lactis* strains. In 38 strains, *tet*(W) was flanked by an IS5-like element and an open reading frame encoding a hypothetical protein, which exhibited a similar GC content (51–53%). These genes were positioned in the same genomic context within the examined genomes. Phylogenetically, the *B. animalis* subsp. *lactis tet*(W) cluster in a clade separate from *tet*(W) of other species and genera. This is not the case for *tet*(W) encoded by other bifidobacteria and other species where *tet*(W) is often found in association with transferable elements or in different genomic regions. An IS5-like element identical to the one flanking the *B. animalis* subsp. *lactis tet*(W) has been found in a human gut related bacterium, but it was not associated with any *tet*(W) genes. This suggests that the IS5-like element is not associated with genetic mobility. *tet*(W) and the IS5 element have previously been shown to be co-transcribed, indicating that co-localization may be associated with *tet*(W) expression. Here, we present a method where phylogenetic and *in silico* genome analysis can be used to determine whether antibiotic resistance genes should be considered innate (intrinsic) or acquired. We find that *B. animalis* subsp. *lactis encoded tet*(W) is part of the ancient resistome and thereby possess a negligible risk of transfer.

## Introduction

Antibiotic resistance genes are widely spread among bacteria and they pose a serious threat to human health as they can compromise our ability to treat bacterial infections (World Health Organisation (WHO), 2017). Although the extensive use of antibiotics to treat infections in both humans and animals is considered to be the main reason for the development and spread of resistance genes (Levy and Bonnie, 2004; WHO, 2011), they have been present long before the introduction of antibiotics to the clinic (Martínez, 2008; Allen et al., 2010). Antibiotics are naturally produced by environmental microorganisms and the producers often have “self-resistance” encoded by antibiotic resistance genes located in the antibiotic biosynthesis gene clusters (Martínez, 2008). Some antibiotic resistance genes show homology to housekeeping genes such as those involved in protein synthesis suggesting that they may have evolved from such functions and this could explain their prevalence among bacteria (Martínez, 2008; Allen et al., 2010). Antibiotic resistance genes have mainly been studied in clinically relevant bacteria and often in relation to horizontally transferable elements (Shrivastava et al., 2018). In contrast, less attention has been paid to antibiotic resistance in non-pathogenic bacteria (Klare et al., 2007; Agersø et al., 2019; Campedelli et al., 2019), e.g., bacteria ingested via the food chain.

When products contain viable, non-pathogenic bacteria, e.g., fermented food, probiotics or feed additives, it is a requirement from legal authorities [e.g., European Food Safety Authority (EFSA)] that these bacteria do not possess acquired genes encoding resistance toward antimicrobials, which are considered as highly or critically important for treatment of humans and/or animals by the World Health Organization (WHO) (WHO, 2011; EFSA panel on Additives and Products or Substances used in Animal Feed (FEEDAP), 2018). However, some bacteria are intrinsically resistant to some of the antimicrobials (Peterson and Kaur, 2018). Impermeability of the outer membrane provides resistance to vancomycin for *Escherichia coli* and other Gram-negative bacteria (Arthur and Courvalin, 1993). *Bacillus licheniformis* and *Bacillus paralicheniformis* are resistant (or reduced in susceptibility) to erythromycin, chloramphenicol and streptomycin due to putative intrinsic resistance genes (Agersø et al., 2019).

Thus, homology to a known antibiotic resistance gene does not in itself indicate whether a putative resistance gene is acquired or intrinsic. Therefore, analysis of the genetic context and comparison to other genomes within the same species/subspecies are needed, although exact guidance on this is not provided by EFSA (EFSA panel on Additives and Products or Substances used in Animal Feed (FEEDAP), 2018).

Tetracyclines are broad spectrum antibiotics, which have been used for treatment of infections in humans and animals since the early 1950s and resistance toward tetracyclines is widespread. The *tet*(W) tetracycline resistance gene encodes a protection protein that attaches to the ribosome and causes an alteration of the ribosomal conformation to which tetracycline cannot bind and therefore protein synthesis can proceed (Chopra and Roberts, 2001; Connell et al., 2003). Genes with more than 80% identity to *tet*(W) have been found in 19 different genera belonging to both Gram-positive and Gram-negative bacteria and thus, it is the most widely spread tetracycline resistance gene class (Chopra and Roberts, 2001). The first *tet*(W) gene was reported in *Butyrivibrio fibrisolvens* located on a Tn *B1230*-like transposable element, which has spread to several different genera due to the broad host range of the element (Scott et al., 1997; Barbosa et al., 1999). Transfer of *tet*(W) in association with mobile genetic elements has also been reported to occur at low frequencies in *Bifidobacterium longum* strain F8 (Kazimierczak et al., 2006), *Arcanobacterium pyogenes* (Billington et al., 2002) and *Streptococcus suis* (Palmieri et al., 2011).

Several bifidobacterial species carry *tet*(W) genes, including *B. longum*, *B. thermophilum* and *B. bifidum* (Ammor et al., 2008). *tet*(W) is widespread and confers a low level of tetracycline resistance in *B. animalis* subsp. *lactis* that varies over three two-fold dilutions between different strains (Gueimonde et al., 2010), which has been suggested to be caused by genetic diversity in the *mia*A gene encoding for a tRNA dimethylallyltransferase (Milani et al., 2013). Furthermore, bile exposure have been shown to induce *tet*(W) expression (Gueimonde et al., 2010). The widespread nature of *tet*(W) suggest that it confers a selective advantage, perhaps a physiological function such as improving translation under the stress conditions of the gut. Although unsuccessful transfer studies are often not published, several studies on transferability of *tet*(W) from *B. animalis* subsp. *lactis* to other bacterial species and genera are published and all were unsuccessful (Gueimonde et al., 2010; Naghizadeh Raeisi et al., 2018; Polit et al., 2018). Bifidobacteria are Gram-positive, anaerobic, non-motile and non-spore-forming bacteria, which are commonly found in the gastrointestinal tract of various animals and humans, the human oral cavity and sewage (Milani et al., 2014). Members of the *Bifidobacterium* genus are among the first microbes to colonize the human gastrointestinal tract of newborns. Multiple health beneficial effects including reduction of diarrhea, colorectal cancer prevention and inhibition of pathogen growth and adherence have been reported for *Bifidobacterium* spp. (Turroni et al., 2012; O’Callaghan and van Sinderen, 2016). Therefore, many *Bifidobacterium* spp. are widely used in probiotic products (Garrigues et al., 2010). *B. animalis* including *B. animalis* subsp. *lactis* have had Qualified Presumption of Safety (QPS) status by EFSA since the establishment of the QPS concept in 2007 (Barlow et al., 2007; Koutsoumanis et al., 2020) and specific strains have acquired the Generally Recognized as Safe (GRAS) status from the Food and Drug Administration (FDA) in the United States (O’Callaghan and van Sinderen, 2016).

The aim of this study was to assess the phylogenetic relationship of *tet*(W) in *B. animalis* subsp. *lactis* through phylogenetic analysis, analysis of the genetic context surrounding the gene and core genome analysis. The study will serve as evidence to further establish that *tet*(W) in *B. animalis* subsp. *lactis* is innate; it originates from the ancestral host and has retained the same genomic position ever since. This supports the common perception that *tet*(W) should be considered an intrinsic and non-transferable gene in *B. animalis* subsp. *lactis*.

## Materials and Methods

### Bacterial Genomes, Subspecies Identification and Genome Quality

All publicly available genome sequences of *B. animalis* subsp. *lactis* (50 strains including the type strain DSM 10140) and *B. animalis* subsp. *animalis* (8 strains including the type strain ATCC 25527) were downloaded from the NCBI microbe genome database on the 21st of November 2019 (Sayers et al., 2019).

Subspecies identification was either obtained from previously published articles (Lugli et al., 2019) or performed by employing the *rpo*A and 16S ribosomal DNA sequence. A >98% identity to the type strain genes was used as threshold and the genes should furthermore be different from the type strain of a related subspecies, in this case *B. animalis* subsp. *animalis*, as shown through a phylogenetic tree (data not shown).

The sequence quality was assessed and sequences with an average coverage of ≥30× and a contig number below 120 were considered acceptable for phylogenetic analysis. The quality of the genomes was also evaluated by checking that the length of the sequenced genome corresponds with the expected length of the genome, based on the type strain (Milani et al., 2014).

Other bifidobacterial species, which have been shown to harbor *tet*(W) (Ammor et al., 2008; Wang et al., 2017) were also downloaded from the NCBI microbe genome database on the 21st of November 2019 and included *B. longum* (14 strains, type strain NCTC11818), *B. thermophilum* (6 strains, type strain DSM 20212), *B. bifidum* (11 strains, type strain ATCC 29521), *B. pseudolongum* (4 strains, type strain DSM 20099), *B. pseudocatenulatum* (3 strains, type strain DSM 20438) and *B. breve* (41 strains, type strain NCTC 11815). All *tet*(W) sequences from other genera where the gene have been described (Scott et al., 1997; Chopra and Roberts, 2001; Flórez et al., 2006; Kazimierczak et al., 2006; Ammor et al., 2008; Palmieri et al., 2011; Schröder et al., 2012) and shared identity to the *tet*(W) gene found in *B. animalis* subsp. *lactis* were also downloaded from NCBI on the 21st of November 2019.

### Screening for *tet*(W), Genome Annotation and Examination of Sequences Flanking *tet*(W)

ResFinder (Zankari et al., 2012), with a 80% identity threshold, was used to search for the presence of *tet*(W) in the examined genomes and the Rapid Annotation using Subsystems Technology (RAST) server with default settings was used to annotate the genomes. The annotated genomes were downloaded in GenBank format from the RAST server (Aziz et al., 2008; Overbeek et al., 2014) and imported to CLC Genomics Workbench 20 (Qiagen Bioinformatics, Aarhus, Denmark), where the presence of *tet*(W), its flanking genes and presence of mobile genetic elements was examined. *tet*(W) nucleotide and protein sequences was extracted from the annotated genomes for further phylogenetic analysis. GC content of *tet*(W) and other genes was assessed by employing the DNA/RNA GC Content Calculator at ENDMEMO (Endmemo, 2020).

### ISFinder

The blastN tool available at ISFinder (Siguier et al., 2006) with default settings was used to determine the identity of the mobile genetic protein next to *tet*(W) in *B. animalis* subsp. *lactis* and its sequence was used to search for its presence in other genomic regions in the *B. animalis* subsp. *lactis* genomes, which was performed in CLC Genomics Workbench 20 (Qiagen Bioinformatics, Aarhus, Denmark).

### *tet*(W) Nucleotide and Amino Acid Phylogenetic Analysis

The phylogenetic analysis of *tet*(W) included both the nucleotide and protein sequences from *B. animalis* subsp. *lactis* (Supplementary Table 1) and *tet*(W) genes found in other bifidobacterial species and other genera where the presence of *tet*(W) previously have been published (Table 1) (Scott et al., 1997; Chopra and Roberts, 2001; Flórez et al., 2006; Kazimierczak et al., 2006; Ammor et al., 2008; Palmieri et al., 2011; Schröder et al., 2012). The nucleotide and protein *tet*(W) sequences was either extracted from the annotated genomes or from NCBI (Sayers et al., 2019).

**TABLE 1:** *tet*(W) encoded by Gram-positive and Gram-negative bacteria.

**Strains**	**Nucleotide identity (%) to *B. animalis* subsp. *lactis* DSM 10140 *tet*(W)**	**Accession number**	**Mobile genetic elements**	**Horizontal transfer confirmed**	**References**
**Gram-positive bacteria**					
***Arcanobacterium pyogenes***					
BBR1	91.79%	AY049983	Integrase, putative mobilization protein, mobilization protein	Yes (18)	Chopra and Roberts, 2001; Billington et al., 2002
***Bifidobacterium bifidum***					
L22	98.01%	EU434755	No MGE		Ammor et al., 2008
***Bifidobacterium breve***					
12L	98.01%	NZ_CP006711	Integrase		NCBI database
139W423	99.74%	CP021556	Transposase, integrase and mobile element protein		Bottacini et al., 2018
lw01	98.06%	CP034192	No MGE		Wang et al., 2019
***Bifidobacterium longum***					
BG7	98.85%	CP010453	Transposase, mobile element protein and phage infection protein		Kwon et al., 2015
BXY01	99.74%	CP008885	Transposases and mobile element proteins		NCBI database
H66	98.06%	DQ060146	No MGE		Flórez et al., 2006
F8	99.37%	DQ294299	Tandem repeat flanking a transposase	Yes (17)	Kazimierczak et al., 2006
L42	98.06%	EU434756	Transposase		Ammor et al., 2008
B93	97.96%	EU434749	NA		Ammor et al., 2008
B94	97.96%	EU434750	NA		Ammor et al., 2008
E111	98.01%	EU434751	NA		Ammor et al., 2008
LMG 13197	99.69%	EU434752	NA		Ammor et al., 2008
***Bifidobacterium thermophilum***					
DSM 20210 (type strain)	99.69%	NZ_JDUB00000000	No MGE		Sun et al., 2015
DSM 20212	99.74%	NZ_JHWM00000000	No MGE		NCBI database
LMG 21813	99.69%	EU434753	No MGE		Ammor et al., 2008
RBL67	99.74%	CP004346	No MGE		Rbl et al., 2013
***Bifidobacterium pseudocatenulatum***					
DSM 20438 (type strain)	99.38%	NZ_AP012330	No MGE		Morita et al., 2015
12	98.01%	CP025199	No MGE		NCBI database
***Bifidobacterium pseudolongum***					
DSM 20092	98.06%	CP017695	Mobile element protein, transposase		NCBI database
***Clostridium difficile***					
CD5	98.85%	AM749838	No MGE		Spigaglia et al., 2008
***Corynebacterium***					
DSM 45100, pJA144188	99.69%	NC_014167	Plasmid		Schröder et al., 2012
***Lactobacillus reuteri***					
PA-16	99.74%	FJ489649	Transposase		Egervärn et al., 2009
ATCC 55730, pLR581	99.63%	EU583804	Plasmid		Egervärn et al., 2010
***Roseburia sp.***					
A2-183	98.01%	AJ421625	Putative mobilization protein		Flórez et al., 2006; Kazimierczak et al., 2006
***Streptococcus suis***					
SsCA-1	98.85%	FN396364	Protein with putative involvement DNA transfer		Chopra and Roberts, 2001; Palmieri et al., 2011
Phi-SsUD	99.69%	FN997652	Genetic element with typical phage organization	Yes (19)	Palmieri et al., 2011
GZ1	99.74%	CP000837	No MGE		Palmieri et al., 2011
***Trueperella pyogenes***					
TP3	98.33%	CP033904	IS21 family transposase, conjugal transfer protein TrbL		Feßler and Schwarz, 2017
**Gram-negative bacteria**					
***Butyrivibrio fibrosolvens***					
Tn 1230	98.06%	AJ222769	Tn1230 transposon	Yes (16)	Scott et al., 1997; Chopra and Roberts, 2001
JK51	98.01%	AJ427421	No MGE		Chopra and Roberts, 2001; Kazimierczak et al., 2006
***Megasphaera elsdenii***					
2–9	No significant similarity found	AY196917	NA		Chopra and Roberts, 2001; Stanton and Humphrey, 2003
7–11	No significant similarity found	AY196919	NA		Chopra and Roberts, 2001; Stanton and Humphrey, 2003
4–13	No significant similarity found	AY196918	NA		Chopra and Roberts, 2001; Stanton and Humphrey, 2003
25–50	98.01%	AY485125	NA		Stanton and Humphrey, 2003
***Mitsuokella multiacidus***					
P208-58	98.06%	AJ427422	No MGE		Chopra and Roberts, 2001; Flórez et al., 2006; Kazimierczak et al., 2006
***Selenomonas ruminantium***					
FB322	99.58%	DQ294295	No MGE		Kazimierczak et al., 2006

*NA, whole genome sequence was not available, the flanking sequences could therefore not be examined. Accession number provided are either nucleotide or genome accession number.*

ClustalX2 (Larkin et al., 2007) was used to perform a pairwise multiple alignment of the *tet*(W) sequences (Higgins and Sharp, 1988) and BioEdit (Hall, 1999) was used to remove gaps and unpaired ends. The nucleotide phylogeny was built by evolutionary analysis by the Maximum Likelihood method and Tamura-Nei model by MEGA X (Tamura and Nei, 1993; Kumar et al., 2018) and the amino acid phylogeny was built by evolutionary analysis by Maximum Likelihood method and JTT matrix-based model also by MEGA X (Jones et al., 1992; Kumar et al., 2018). Number of single nucleotide polymorphisms (SNPs) and single amino acid polymorphisms (SAPs) was obtained from the multiple alignment output from MEGA X that was used to build the phylogenetic relationships.

### Core Genome Phylogeny

The genomes, either fully assembled or contigs were annotated by Prokka, which annotates genomes through the use of different tools including Prodigal (coding sequences), RNAmmer (Ribosomal RNA genes), Aragorn (Transfer RNA genes), SignalP (Signal leader peptides) and Infernal (Non-coding RNA) (Seemann, 2014). Prokka annotation is a requirement for using Roary, since the .gff file (file containing sequences and annotations) provided by Prokka is used by Roary to create a multi-FASTA alignment of all the core genes (Page et al., 2015). Roary was set to perform nucleotide alignment using MAFFT and a Blastp percentage identity at 80% (Katoh, 2002). FastTree was used to produce an approximately maximum-likelihood phylogenetic tree from the core gene alignment file, which was visualized by MEGA X (Price et al., 2009, 2010; Kumar et al., 2018).

## Results and Discussion

### Assessment of Genome Quality

A total of 50 publicly available *B. animalis* subsp. *lactis* strains including the type strain DSM 10140 were downloaded from NCBI and consisted either of contigs or assembled genomes (Supplementary Table 1). The sequence quality was assessed and sequences with an average coverage of ≥30 fold and a contig number below 120 were considered acceptable. On this basis, six strains (B420, DS1_2, BI-04, IDCC4301, CF3_2, AD011) were excluded from the study. The genomes of CNCM I-2994 (Chervaux et al., 2011) and AD011 (Kim et al., 2009) had both been sequenced by Sanger shotgun sequencing and consist of complete genomes. However, AD011 has previously been shown to exhibit a poor sequence quality and was therefore excluded (Garrigues et al., 2010), CNCM I-2994 was not excluded from the study. A total of 44 genome sequences were therefore acceptable for further phylogenetic analysis.

The *B. animalis* subsp. *lactis* genomes exhibited a size of 1.91–2.08 Mb with a GC content of 60.0–60.6% (Supplementary Table 1), which is in agreement with data for the type strain of the subspecies (Milani et al., 2014).

Subspecies identification was either obtained from previously published articles (Lugli et al., 2019) or performed by analysis of the *rpo*A and 16S ribosomal DNA sequence.

### Diversity of the *B. animalis* subsp. *lactis* Genomes

The majority of the *B. animalis* subsp. *lactis* strains originated from human feces, but also from food samples, dietary supplements and domestic pigs, chimpanzees, rabbits, vervet monkeys, a barbary macaque, three different dog breeds and one strain, the genomic unique ATCC 27673 (Loquasto et al., 2013) originated from sewage (Supplementary Table 1). Species within the bifidobacterial genera are commonly found in the gastrointestinal tract of various animals, the human oral cavity and sewage (Milani et al., 2014) and the strains in this study therefore represent the most common habitats of bifidobacteria.

Since *B. animalis* subsp. *lactis* is included in a wide range of probiotics, it cannot be excluded that the strains isolated from human feces, domestic pigs and dogs originate from ingested products such as probiotics. However, the strain collection also include strains such as Bl12 that has been isolated from a healthy patient, which has not ingested probiotic products (Milani et al., 2013) and rabbits and monkeys have with high likelihood not been exposed to probiotics and these strains are therefore expected to be diverse from the industrially exploited strains. The genome sizes of the different strains also vary, which also indicate that the strains are diverse (Supplementary Table 1). Most of the strains are isolated or submitted to NCBI between year 2006–2018, which reflect the increased focus on probiotics in the last decades (Gogineni, 2013), while the type strain DSM 10140 originates from 1997 (Supplementary Table 1). However, the submission date of the genome sequences to NCBI does not necessarily reflect the time of isolation as some strains are isolated even earlier.

*B. animalis* subsp. *lactis* has previously been shown to be a strict monophyletic bifidobacterial taxon that has recently evolved (Milani et al., 2013), however, some diversity is observed between the strains within the subspecies based on the presence of truly unique genes in some of the strains (Lugli et al., 2019). The strains with the highest number of truly unique genes are also included in this study. It is therefore concluded that the strains included in the current study represent the diversity within the subspecies.

### The *tet*(W) Gene and its Genomic Location in *B. animalis* subsp. *lactis*

**FIGURE 1:**
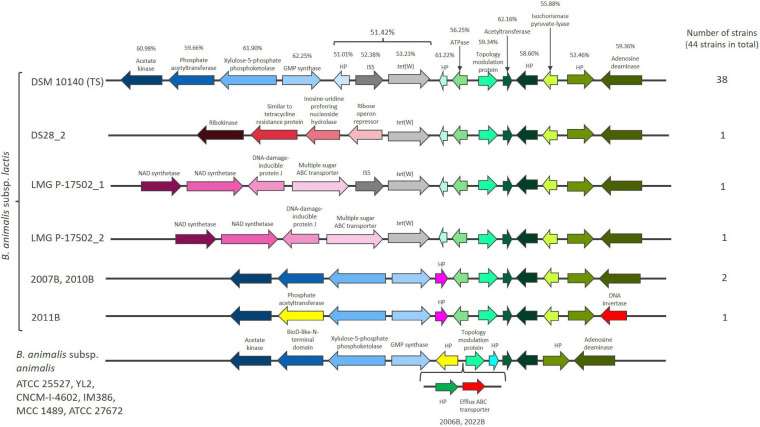
The chromosomal region flanking *tet*(W) in *Bifidobacterium animalis* subsp. *lactis* and the same region in *Bifidobacterium animalis* subsp. *animalis.* Hypothetical proteins are designated HP. GC content (%) is provided for the genes found in the *B. animalis* subsp. *lactis* type strains (TS) DSM 10140. Genes that are present in the majority of the examined *B. animalis* subsp. *lactis* strains (represented by DSM 10140) has the same color in all the shown strains [blue colors downstream of *tet*(W) and green colors upstream of *tet*(W)].

A 1920 bp *tet*(W) gene flanked by genes annotated as mobile element protein (966 bp), with inverted repeats at both ends of 50 bp and a hypothetical protein (HP) of unknown function (183 bp) was found in the majority of the studied *B. animalis* subsp. *lactis* strains (38 out of 44). These genes exhibit similar GC content (51.01–53.23%), which is lower than the flanking genes in the genetic region (52.46–62.25%) (Figure 1) and the average of the genome (60.0–60.6%) (Supplementary Table 1). *tet*(W) genes found in non-bifidobacterial and bifidobacterial species exhibit a GC content of 52.19–53.18%, indicating that *tet*(W) genes generally exhibit a GC content around 53%.

The three strains originating from dogs (2007B, 2010B, 2011B) did not encode *tet*(W), the mobile element protein or the HP (Figure 1 and Supplementary Table 1). Two strains (DS28_2, LMG P-17502_2) only encoded the *tet*(W) gene, while LMG P-17502 encoded *tet*(W) and the mobile element protein (Figure 1). UBBLa 70 exhibited a large deletion in the *tet*(W), with only 117 bp remaining and two strains (ATCC 27673, 1528B) encoded a truncated version of the mobile element protein. This indicate that the three genes have been present originally in *B. animalis* subsp. *lactis* but have been subject to deletion in some strains. Despite these differences, the presence of *tet*(W), the putative mobile element protein and the HP are highly conserved within *B. animalis* subsp. *lactis* strains. This conservation was even observed in the strains that are more genomic unique which include ATCC 27673 and 1528B, and the Bl12 strain and the strains isolated from monkeys and rabbits. This suggest that the genetic organization surrounding *tet*(W) is not only present in the industrially exploited *B. animalis* subsp. *lactis* strains.

The *tet*(W), the mobile element protein and the HP genes were positioned in the same genomic context in the majority of the examined strains, however, in a few strains, alterations downstream (DS28_2, LMG P-17502_1, LMG P-17502_2, 2007B, 2010B, 2011B) and upstream (2011B) (Figure 1) of the three genes were observed. These were the same strains that exhibited complete or partial deletions of the *tet*(W), the mobile element protein and HP genes.

The genomic position of *tet*(W) was also reported by Rozman et al. (2020). They suggest that *tet*(W) and its flanking genes from the HP before the IS element to the HP after isochorismate pyruvate-lyase (Figure 1), based on nucleotide bias and codon usage bias, is part of a putative genomic island that has co-evolved together with *B. animalis* subsp. *lactis* and originate from an ancestral host (Guo et al., 2012; Bertelli et al., 2017). The codon usage bias corresponds with the gene GC content being lower in these genes compared to the rest of the genome. Genomic islands are defined as clusters of genes in bacterial genomes of probable horizontal origin and they often provide adaptive traits that has the ability to enhance the fitness of bacteria within a specific niche (Dobrindt et al., 2004). The putative genomic island in *B. animalis* subsp. *lactis* encodes for genes involved in cell metabolism and gene regulation and has not been found in other bacteria (Rozman et al., 2020). This could suggest that the putative genomic island including *tet*(W) encodes for important *B. animalis* subsp. *lactis* niche factors, which enable it to survive and compete for nutrients in the gut and has been part of the genome of *B. animalis* subsp. *lactis* long before the antibiotic era.

The *tet*(W), the mobile element protein and the HP gene were absent in all eight *B. animalis* subsp. *animalis* strains included in the study (Supplementary Table 1), which otherwise exhibited almost identical gene organization in the genomic region including the genes part of the putative genomic island (Figure 1). This could suggest that the *tet*(W), the mobile element protein and HP genes have been inserted in an ancestor of the *B. animalis* subsp. *lactis* close to subspecies differentiation and most likely lost by the three dog originating strains (2007B, 2010B, 2011B) not carrying *tet*(W).

### Identification of the Putative Mobile Element Protein Flanking *tet*(W)

The presence of a putative mobile element protein next to *tet*(W) has previously been reported (Ammor et al., 2008; Gueimonde et al., 2010; Rozman et al., 2020). The sequence encodes a putative DDE transposase gene that is flanked by inverted repeats upstream and downstream of 50 bp, which collectively belong to the insertion sequence (IS) 5-like element ISBian1 family that originate from *B. animalis* according to ISFinder (Siguier et al., 2006).

DDE transposases are able to catalyze the movement of IS elements and transposons by introducing nicks at each end of the elements (Frost et al., 2005) and are able to move within a genome or horizontally if they are part of mobile genetic element vectors such as plasmids, conjugative transposon and phages (Vandecraen et al., 2017). However, several studies have been unsuccessful in transferring *tet*(W) from *B. animalis* subsp. *lactis* to other species and genera (Gueimonde et al., 2010; Naghizadeh Raeisi et al., 2018; Polit et al., 2018), A BLASTp analysis showed that the IS5-like element ISBian1 family with 99.07% identity was found in the human ileum isolated *Angelaksiella massiliensis* (Mailhe et al., 2017) and the IS5 element was not associated with *tet*(W) in this species. The IS5 element was not found in other bifidobacterial species besides *B. animalis* subsp. *lactis*. The IS5 element was not found in other positions within the *B. animalis* subsp. *lactis* genomes and the inverted repeats flanking the transposase was only flanking the transposase next to *tet*(W). This indicates that the IS element is stably positioned next to *tet*(W) and does not mobilize within the *B. animalis* subsp. *lactis* genome, which is in accordance with the stable nature of the *B. animalis* subsp. *lactis* genome (Morovic et al., 2018).

Besides IS elements involvement in mobilization, IS5 elements are mainly able to modulate the expression of neighboring genes through co-transcription from the transposase promoter located in the terminal inverted repeat if inserted into non-coding regions (Schnetz and Rak, 1992; Luque et al., 2006; Vandecraen et al., 2017). The IS5 element flanking *tet*(W) in *B. animalis* subsp. *lactis* is positioned in a non-coding region meaning it does not cause deletion of other genes (Figure 1) and has previously been shown to be co-transcribed with *tet*(W) (Gueimonde et al., 2010). This indicates that the IS5 element potentially is involved in modulating the expression of *tet*(W) rather than mobilization.

### *tet*(W) Encoded by Gram-Positive and Gram-Negative Bacteria

All previously published *tet*(W) genes were included in the analysis. Direct submissions at NCBI also include other *tet*(W) genes, however, none of these exhibited 100% identity to the subspecies *B. animalis* subsp. lactis *tet*(W) and we did not find any variants not represented in the analysis (data not shown). The published *tet*(W) genes are therefore a good presentation of *tet*(W).

*tet*(W) is one of the most widely spread resistance genes and is both found in Gram-positive and -negative bacteria (Chopra and Roberts, 2001). Despite the wide spread nature of *tet*(W), it was not found to be encoded by all the strains within the examined Gram-positive and -negative species, showing that *tet*(W) has been acquired by a few strains or lost as compared with *B. animalis* subsp. *lactis* where it is a general genetic feature of the subspecies. For both the Gram-positive and -negative bacteria other than *B. animalis* subsp. *lactis, tet*(W) was often found to be flanked by mobile genetic elements (Table 1) and in some strains *tet*(W) was positioned in a genomic region with several mobile genetic elements, e.g., *B. longum* BG7 and *A. pyogenes* BBR1. Transfer of *tet*(W) has been reported for *B. longum* strain F8 (Kazimierczak et al., 2006), *A. pyogenes* (Billington et al., 2002), *S. suis* (Palmieri et al., 2011) and *B. fibrosolvens* (Scott et al., 1997). Within species, the *tet*(W) genes in the examined Gram-positive and -negative bacteria were positioned in different genomic regions. Together, this indicates that *tet*(W) probably has been acquired independently in the examined bacteria in Table 1.

The observation that *tet*(W) is generally present in *B. animalis* subsp. *lactis* strains and is positioned in the same genomic region indicates that *tet*(W) is conserved and thereby an innate part of the subspecies, while *tet*(W) only has been acquired by a few strains within the examined Gram-positive and -negative bacterial species.

### *tet*(W) Encoded by *B. animalis* subsp. *lactis* Is Distinct From *tet*(W) Encoded by Other Bacteria

**FIGURE 2:**
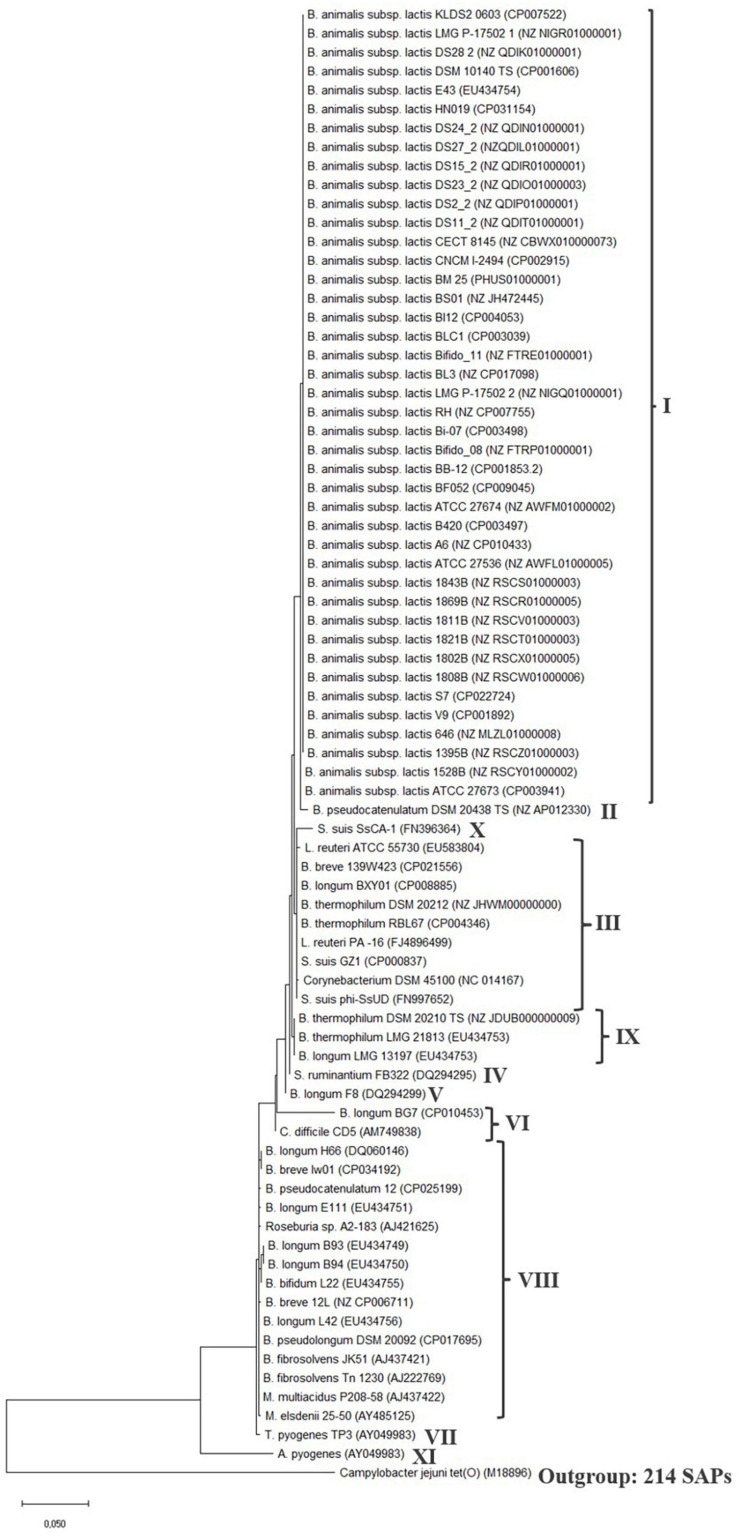
*tet*(W) protein phylogenetic tree. The tree was built by evolutionary analysis by maximum likelihood method and JTT matrix-based model (Jones et al., 1992; Kumar et al., 2018). The branch lengths are measured in the number of substitutions per site. Strain name and genome or *tet*(W) gene accession number is provided for the sequences. Type strains (TS) are included for the species, when the type strain encodes *tet*(W). Clades are defined by the number of SAPs, which can be seen in Table 2. The phylogenetic tree was rooted with the ribosomal protection gene *tet*(O) from *Campylobacter jejuni* (M18896) as an outgroup and similar results was obtained with the *Streptococcal* ribosomal protection gene *tet*(M) (X04388) (data not shown) (Levy et al., 1999).

A phylogenetic analysis was conducted of the *tet*(W) gene (Supplementary Figure 1) and protein (Figure 2) present in *B. animalis* subsp. *lactis* (Supplementary Table 1) and in the examined Gram-positive and -negative bacteria (Table 1).

**TABLE 2:** Clades in the nucleotide and protein phylogenetic trees based on number of SNPs and SAPs.

**Clades**	**SNPs**	**SAPs**	**Species**
**I**	0–1	0–1	*Bifidobacterium animalis* subsp. *lactis*
**II**	12	5	*Bifidobacterium pseudocatenulatum*
**III**	11–13	5–7	*Bifidobacterium breve, Bifidobacterium longum, Bifidobacterium thermophilum, Streptococcus suis, Corynebacterium, Lactobacillus reuteri*
**IV**	15	6	*Selenomonas ruminantium*
**V**	19	8	*Bifidobacterium longum*
**VI**	26–29	15	*Bifidobacterium longum, Clostridium difficile*
**VII**	38	20	*Trueperella pyogenes*
**VIII**	44–46	21–23	*Bifidobacterium bifidum, Bifidobacterium breve, Bifidobacterium longum, Bifidobacterium pseudolongum, Bifidobacterium pseudocatenulatum, Butyrivibrio fibrosolvens, Mitsuokella multicidus*, *Megasphaera elsdenii*, *Roseburia* sp.
**IX**	13	6	*Bifidobacterium longum, Bifidobacterium thermophilum*
**X**	28	13	*Streptococcus suis*
**XI**	161	69	*Arcanobacterium pyogenes*

The *tet*(W) genes encoded by the *M. elsdenii* strains (2–9, 7–11, 4–13) was shorter (1474–1476 bp) and exhibited a GC content (54.61–55.22%) higher compared to the other examined *tet*(W) genes and was therefore excluded from the phylogenetic analysis. The *tet*(W) gene of the remaining *M. elsdenii* strain (25–50) was found to be more similar to the other *tet*(W) genes and therefore included in the analysis.

Generally, the phylogenetic trees showed a high similarity between the different *tet*(W) genes and proteins, which is in agreement with previous observations (Aminov and Mackie, 2007), with the number of SNPs ranging from 1 to 46 and single amino acid polymorphisms (SAPs) ranging from 1 to 23 in the coding region compared to the *tet*(W) genes encoded by *B. animalis* subsp. *lactis.* The *tet*(W) gene encoded by *A. pyogenes* differed the most from *B. animalis* subsp. *lactis tet*(W) (161 SNPs and 69 SAPs). None of the SNPs lead to a premature stop codon. Based on the number of SNPs and SAPs (Table 2), clades were formed in the phylogenetic trees (Figure 2 and Supplementary Figure 1), which follows the phylogeny for *B. animalis* subsp. *lactis* but not the other examined Gram-positive and -negative bacteria.

The phylogenetic analysis showed that the *tet*(W) genes (Supplementary Figure 1) and proteins (Figure 2) from the *B. animalis* subsp. *lactis* strains share a high degree of homology and forms a separate clade.

The *tet*(W) gene and protein in the *B. pseudocatenulatum* type strain DSM 20438 (Genome GC content 56.40%) was located nearest the *B. animalis* subsp. *lactis tet*(W) genes and proteins in the phylogenetic trees and exhibited 12 SNPs and 5 SAPs compared to the *tet*(W) genes and proteins encoded by *B. animalis* subsp. *lactis*. The *tet*(W) gene encoded by *B. pseudocatenulatum* DSM 20438 and *B. animalis* subsp. *lactis* both exhibit a high identity to *tet*(W) from *S. suis* (FN396364). The *tet*(W) gene encoded by *B. pseudocatenulatum* strain 12 exhibited 45 SNPs and 22 SAPs and was located in another clade than the DSM 20438 *tet*(W) gene, indicating that the *tet*(W) encoded by the two *B. pseudocatenulatum* strains differ. *tet*(W) has been shown to be present in 33–41% of *B. pseudocatenulatum* isolates from human (Aires et al., 2007; Wang et al., 2017), no mobile genetic elements including IS5 elements was found in the flanking regions of *tet*(W) in the two examined strains (Table 1) and transfer of *tet*(W) from *B. pseudocatenulatum* have so far not been shown to occur (Wang et al., 2017). An examination of the flanking sequences of *tet*(W) in *B. pseudocatenulatum* type strain DSM 20438 revealed that the downstream genes were organized similarly as the genes downstream of *tet*(W) in the majority of the studied *B. animalis* subsp. *lactis* strains (Figure 1), except that six hypothetical proteins was present between *tet*(W) and the GMP synthase gene and no IS5-like element was present (Supplementary Figure 2). These genes were also present in *B. pseudocatenulatum* strain 12 but in another genetic location than *tet*(W), and in a *B. pseudocatenulatum* strain (ca_0067, NZ_RCXS00000000) that did not encode *tet*(W). This indicates that the presence of these genes is independent of the presence of *tet*(W) and are shared genes between *B. animalis* subsp. *lactis* and *B. pseudocatenulatum*.

The *tet*(W) genes present in the examined Gram-positive and -negative bacteria including the two *B. pseudocatenulatum* strains, were scattered over different clades in the phylogenetic tree indicating that the *tet*(W) genes encoded by these bacteria are diverse, does not follow the phylogeny of the specific species and thereby support the acquired nature of these *tet*(W) genes.

### *tet*(W) Encoded by *B. animalis* subsp. *lactis* Follows the Phylogeny of the Subspecies

**FIGURE 3:**
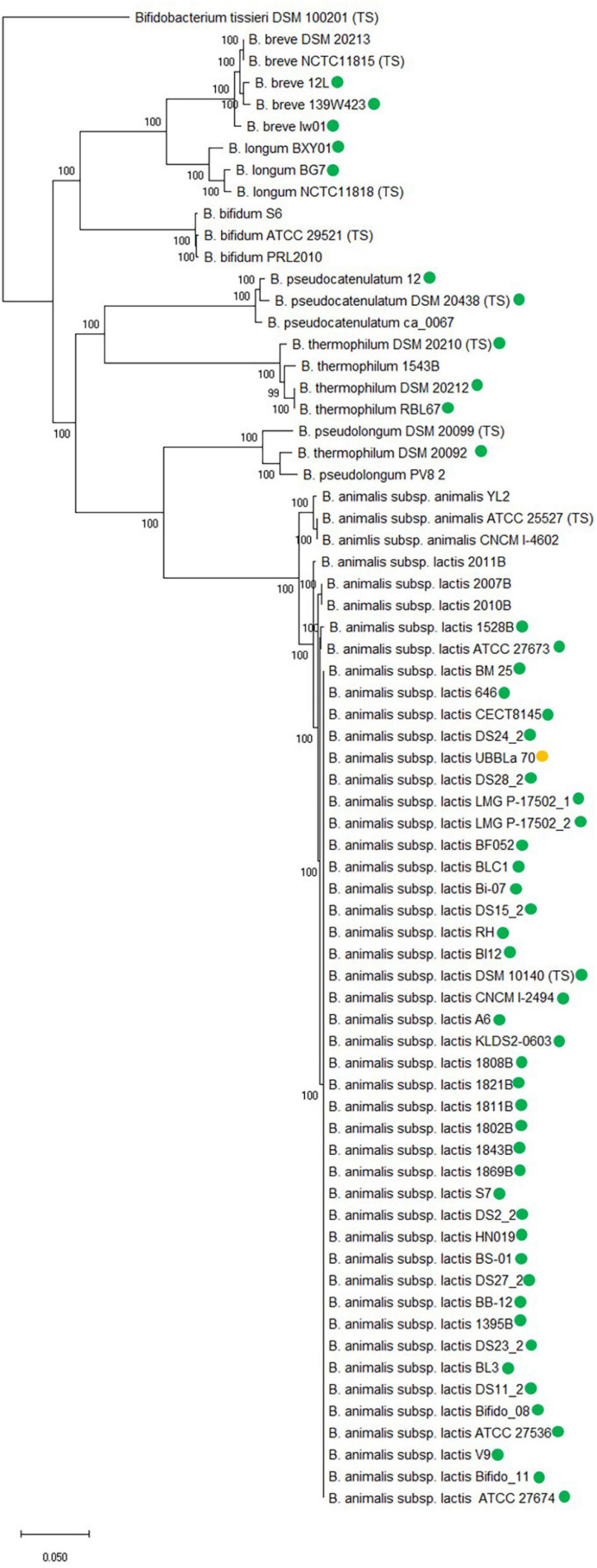
Core genome phylogenetic tree based on 250 core genes which include *B. animalis* subsp. *lactis* strains and other related *Bifidobacterium* species. Type strain has been included for each species, designated TS and strains both with and without *tet*(W) are included for each species, except for *B. animalis* subsp. *animalis* and *B. bifidum*. *tet*(W) positive strains are marked with a green circle. *B. animalis* subsp. *lactis* UBBLa 70 exhibit a *tet*(W) gene with large deletions and is marked with a yellow circle. The tree is rooted with the *Bifidobacterium tissieri* type strain DSM 100201 as an outgroup (Lugli et al., 2018). Bootstrap percentages are shown at node points.

A core genome phylogenetic analysis was conducted with the examined B. animalis subsp. lactis strains (Supplementary Table 1), the bifidobacterial species from Table 1 and B. animalis subsp. animalis strains from Supplementary Table 1 (Figure 3). For each species, strains were included that both did and did not encode *tet*(W), except for *B. animalis* subsp. *animalis* and *B. bifidum*.

The core genome phylogenetic analysis showed that the bifidobacterial species separated from each other in individual clades and both strains with and without *tet*(W) clustered together within species, showing that the core genome analysis was able to separate at species and subspecies level.

The fact that the *tet*(W) gene encoded by the examined *B. animalis* subsp. *lactis* strains formed a separate clade in the gene and protein phylogenetic analysis (Supplementary Figure 1 and Figure 2) similar to the one formed in the core genome phylogenetic tree shows that the phylogeny of *tet*(W) follows the phylogenetic relationship of the subspecies, indicates that *tet*(W) originates from an ancestral host. This is further supported by the gene being positioned in the same genomic context in the examined strains. For the other examined bifidobacterial species, the *tet*(W) genes does not follow the phylogeny of the species, indicating that the *tet*(W) gene has been acquired at different timepoints, which is in line with them being flanked by different mobile genetic elements and positioned in different genomic contexts. This indicates that *tet*(W) present in *B. animalis* subsp. lactis is distinct from *tet*(W) found in other bifidobacterial species and other genera.

## Conclusion

The paper presents a method where *in silico* genome analysis together with phylogenetic analysis can be used to determine whether a gene is innate and thereby not considered a safety concern.

A phylogenetic analysis of *tet*(W) in *B. animalis* subsp. *lactis*, a widely used probiotic bacterium, was performed and shows that *tet*(W) in this specific subspecies is present in the majority of the strains (41 out of 44), positioned in the same genomic region and is different on the amino acid level from *tet*(W) genes found in other species. *tet*(W) is flanked by an IS5-like element, which is known to be present in other human gut related bacteria, however, the IS5-like element was not associated with *tet*(W) in these bacteria. Previously results show that *tet*(W) is co-transcribed with the IS5 transposase in *B. animalis* subsp. *lactis*, indicating that the expression of *tet*(W) is regulated by the IS5 transposase. Together with the previous unsuccessful attempts to transfer *tet*(W), our data suggest that *tet*(W) is non-transferable and that the flanking IS5 element is not involved in mobilization of *tet*(W). The phylogenetic analysis showed that *tet(*W) follows the phylogenetic relationship of the subspecies and is distinct from *tet*(W) found in other genera and bifidobacterial species.

We conclude that *tet*(W) in *B. animalis* subsp. *lactis* originates from an ancestral host and is therefore an innate part of the subspecies and should be considered as innate (intrinsic) in this subspecies. There is therefore a negligible risk that *tet*(W) from *B. animalis* subsp. *lactis* will add to the pool of mobile resistance genes and thus potentially cause treatment failures in humans and animals.

## Data Availability Statement

The original contributions presented in the study are included in the article/Supplementary Material, further inquiries can be directed to the corresponding author.

## Author Contributions

KN-M wrote the manuscript, made figures, tables, performed the analysis and was involved in developing the concept and the method. CS was involved in developing the concept, guiding the analysis, discussion, and review and editing. HI was involved in developing the concept, discussion, and review and editing. YA was involved in conceiving the idea, developing and guiding the concept, analysis, design, discussion, and review and editing. All authors have read and approved the submitted manuscript.

## Conflict of Interest

Most authors were employees at Chr. Hansen A/S, a company that produces strains for plant protection, animal and human health as well as for the food and dairy industry. Some of the authors are share-holders in Chr. Hansen A/S. This does not alter our adherence to Frontiers Microbiology policies on sharing data and materials.

## References

[B1] Agersø Y. Bjerre K. Brockmann E. Johansen E. Nielsen B. Siezen R. (2019). Putative antibiotic resistance genes present in extant Bacillus licheniformis and *Bacillus paralicheniformis* strains are probably intrinsic and part of the ancient resistome. *PLoS One* 14:e0210363. 10.1371/journal.pone.0210363 30645638 PMC6333372

[B2] Aires J. Doucet-Populaire F. Butel M. J. (2007). Tetracycline resistance mediated by tet(W), tet(M), and tet(O) genes of Bifidobacterium isolates from humans. *Appl. Environ. Microbiol.* 73 2751–2754. 10.1128/aem.02459-06 17308188 PMC1855585

[B3] Allen H. K. Donato J. Wang H. H. Cloud-Hansen K. A. Davies J. Handelsman J. (2010). Call of the wild: Antibiotic resistance genes in natural environments. *Nat. Rev. Microbiol.* 8 251–259. 10.1038/nrmicro2312 20190823

[B4] Aminov R. I. Mackie R. I. (2007). Evolution and ecology of antibiotic resistance genes. *FEMS Microbiol. Lett.* 271 147–161. 10.1111/j.1574-6968.2007.00757.x 17490428

[B5] Ammor M. S. Flórez A. B. Álvarez-Martín P. Margolles A. Mayo B. (2008). Analysis of tetracycline resistance tet (W) genes and their flanking sequences in intestinal *Bifidobacterium* species. *J. Antimicrob. Chemother.* 62 688–693. 10.1093/jac/dkn280 18614524

[B6] Arthur M. Courvalin P. (1993). Genetics and mechanisms of glycopeptide resistance in enterococci. *Antimicrob. Agents Chemother.* 37 1563–1571. 10.1128/aac.37.8.1563 8215264 PMC188020

[B7] Aziz R. K. Bartels D. Best A. DeJongh M. Disz T. Edwards R. A. (2008). The rast server: rapid annotations using subsystems technology. *BMC Genom.* 9:75. 10.1186/1471-2164-9-75 18261238 PMC2265698

[B8] Barbosa T. M. Scott K. P. Flint H. J. (1999). Evidence for recent intergeneric transfer of a new tetracycline resistance gene, tet(W), isolated from Butyrivibrio fibrisolvens, and the occurrence of tet(O) in ruminai bacteria. *Environ. Microbiol.* 1 53–64. 10.1046/j.1462-2920.1999.00004.x 11207718

[B9] Barlow S. Chesson A. Collins J. D. Dybing E. Flynn A. Fruijtier-C. (2007). Introduction of a qualified presumption of safety (QPS) approach for assessment of selected microorganisms referred to efsaopinion of the scientific committee. *EFSA J.* 5 1–16.

[B10] Bertelli C. Laird M. R. Williams K. P. Lau B. Y. Hoad G. Winsor G. L. (2017). IslandViewer 4: expanded prediction of genomic islands for larger-scale datasets. *Nucleic Acids Res.* 45 W30–W35. 28472413 10.1093/nar/gkx343 PMC5570257

[B11] Billington S. J. Songer J. G. Jost B. H. (2002). Widespread distribution of a tet W determinant among tetracycline-resistant isolates of the animal pathogen Arcanobacterium pyogenes. *Antimicrob. Agents Chemother.* 46 1281–1287. 10.1128/aac.46.5.1281-1287.2002 11959557 PMC127165

[B12] Bottacini F. Morrissey R. Roberts R. J. James K. Van Breen J. Egan M. (2018). Comparative genome and methylome analysis reveals restriction/modification system diversity in the gut commensal Bifidobacterium breve. *Nucleic Acids Res.* 46 1860–1877. 10.1093/nar/gkx1289 29294107 PMC5829577

[B13] Campedelli I. Mathur H. Salvetti E. Clarke S. Rea M. C. Torriani S. (2019). Genus-wide assessment of antibiotic resistance in *Lactobacillus* spp. *Appl. Environ. Microbiol.* 85 1–21. 10.1128/AEM.01738-18 PMC6293106 30366997

[B14] Chervaux C. Grimaldi C. Bolotin A. Quinquis B. Legrain-Raspaud S. van Hylckama Vlieg J. E. T. (2011). Genome sequence of the probiotic strain *Bifidobacterium animalis* subsp. lactis CNCM I-2494. *J. Bacteriol.* 193 5560–5561. 10.1128/jb.05716-11 21914878 PMC3187456

[B15] Chopra I. Roberts M. (2001). Tetracycline antibiotics: mode of action, applications, molecular biology, and epidemiology of bacterial resistance. *Microbiol. Mol. Biol. Rev.* 65 232–260. 10.1128/mmbr.65.2.232-260.2001 11381101 PMC99026

[B16] Connell S. R. Tracz D. M. Nierhaus K. H. Taylor D. E. (2003). Ribosomal protection proteins and their mechanism of tetracycline resistance. *Antimicrob. Agents Chemother.* 47 3675–3681. 10.1128/aac.47.12.3675-3681.2003 14638464 PMC296194

[B17] Dobrindt U. Hochhut B. Hentschel U. Hacker J. (2004). Genomic islands in pathogenic and environmental microorganisms. *Nat. Rev. Microbiol.* 2 414–424. 10.1038/nrmicro884 15100694

[B18] EFSA panel on Additives and Products or Substances used in Animal Feed (FEEDAP). (2018). Guidance on the characterisation of microorganisms used as feed additives or as production organisms. *EFSA J.* 16:e05206 32625840 10.2903/j.efsa.2018.5206 PMC7009341

[B19] Egervärn M. Lindmark H. Olsson J. Roos S. (2010). Transferability of a tetracycline resistance gene from probiotic *Lactobacillus reuteri* to bacteria in the gastrointestinal tract of humans. *Antonie van Leeuwenhoek Int. J. Gen. Mol. Microbiol.* 97 189–200. 10.1007/s10482-009-9401-0 19997864

[B20] Egervärn M. Roos S. Lindmark H. (2009). Identification and characterization of antibiotic resistance genes in *Lactobacillus reuteri* and *Lactobacillus plantarum*. *J. Appl. Microbiol.* 107 1658–1668. 19457037 10.1111/j.1365-2672.2009.04352.x

[B21] Endmemo. (2020). *DNA/RNA GC Content Calculator.* Available online at: http://www.endmemo.com/bio/gc.php

[B22] Feßler A. T. Schwarz S. (2017). Antimicrobial Resistance in *Corynebacterium* spp., *Arcanobacterium* spp., and Trueperella pyogenes. *Microbiol. Spectr.* 5. 10.1128/microbiolspec.arba-0021-2017 PMC11687552 29219109

[B23] Flórez A. B. Ammor M. S. Álvarez-Martín P. Margolles A. Mayo B. (2006). Molecular analysis of tet(W) gene-mediated tetracycline resistance in dominant intestinal Bifidobacterium species from healthy humans. *Appl. Environ. Microbiol.* 72 7377–7379. 10.1128/aem.00486-06 16936047 PMC1636146

[B24] Frost L. S. Leplae R. Summers A. O. Toussaint A. (2005). Mobile genetic elements: the agents of open source evolution. *Nat. Rev. Microbiol.* 3 722–732. 10.1038/nrmicro1235 16138100

[B25] Garrigues C. Johansen E. Pedersen M. B. (2010). Complete genome sequence of *Bifidobacterium* animalis subsp. lactis BB-12, a widely consumed probiotic strain. *J. Bacteriol.* 192 2467–2468. 10.1128/jb.00109-10 20190051 PMC2863482

[B26] Gogineni V. K. (2013). Probiotics: history and evolution. *J. Anc. Dis. Prev. Remedies* 1 1–7. 10.1007/978-94-011-2364-8_1

[B27] Gueimonde M. Flórez A. B. Van Hoek A. H. A. M. Stuer-Lauridsen B. Strøman P. De Los Reyes-Gavilán C. G. (2010). Genetic basis of tetracycline resistance in *Bifidobacterium* animalis subsp. lactis. *Appl. Environ. Microbiol.* 76 3364–3369. 10.1128/aem.03096-09 20348299 PMC2869156

[B28] Guo F. B. Wei W. Wang X. L. Lin H. Ding H. Huang J. (2012). Co-evolution of genomic islands and their bacterial hosts revealed through phylogenetic analyses of 17 groups of homologous genomic islands. *Genet. Mol. Res.* 11 3735–3743. 10.4238/2012.october.15.5 23096693

[B29] Hall T. A. (1999). BioEdit: a user-friendly biological sequence alignment editor and analysis program for Windows 95/98/NT. *Nucleic Acids Symp. Ser.* 41 95–98.

[B30] Higgins D. G. Sharp P. M. (1988). CLUSTAL: a package for performing multiple sequence alignment on a microcomputer. *Gene* 73 237–244. 10.1016/0378-1119(88)90330-7 3243435

[B31] Jones D. T. Taylor W. R. Thornton J. M. (1992). The rapid generation of mutation data matrices from protein sequences. *Comput. Appl. Biosci.* 8 275–282. 10.1093/bioinformatics/8.3.275 1633570

[B32] Katoh K. (2002). MAFFT: a novel method for rapid multiple sequence alignment based on fast fourier transform. *Nucleic Acids Res.* 30 3059–3066. 10.1093/nar/gkf436 12136088 PMC135756

[B33] Kazimierczak K. A. Flint H. J. Scott K. P. (2006). Comparative analysis of sequences flanking tet(W) resistance genes in multiple species of gut bacteria. *Antimicrob. Agents Chemother.* 50 2632–2639. 10.1128/aac.01587-05 16870752 PMC1538676

[B34] Kim J. F. Jeong H. Yu D. S. Choi S. H. Hur C. G. Park M. S. (2009). Genome sequence of the probiotic bacterium bifidobacterium animalis subsp. lactis AD011. *J. Bacteriol.* 191 678–679. 10.1128/jb.01515-08 19011029 PMC2620821

[B35] Klare I. Konstabel C. Werner G. Huys G. Vankerckhoven V. Kahlmeter G. (2007). Antimicrobial susceptibilities of Lactobacillus, Pediococcus and Lactococcus human isolates and cultures intended for probiotic or nutritional use. *J. Antimicrob. Chemother.* 59 900–912. 10.1093/jac/dkm035 17369278

[B36] Koutsoumanis K. Allende A. Alvarez-Ordóñez A. Bolton D. Bover-Cid S. Chemaly M. (2020). Update of the list of QPS-recommended biological agents intentionally added to food or feed as notified to EFSA 11: suitability of taxonomic units notified to EFSA until September 2019. *EFSA J.* 18:5965. 10.2903/j.efsa.2020.5965 PMC7448003 32874211

[B37] Kumar S. Stecher G. Li M. Knyaz C. Tamura K. (2018). MEGA X: Molecular evolutionary genetics analysis across computing platforms. *Mol. Biol. Evol.* 35 1547–1549. 10.1093/molbev/msy096 29722887 PMC5967553

[B38] Kwon S. K. Kwak M. J. Seo J. G. Chung M. J. Kim J. F. (2015). Complete genome sequence of Bifidobacterium longum KCTC 12200BP, a probiotic strain promoting the intestinal health. *J. Biotechnol.* 214 169–170. 10.1016/j.jbiotec.2015.09.039 26439427

[B39] Larkin M. A. Blackshields G. Brown N. P. Chenna R. Mcgettigan P. A. McWilliam H. (2007). Clustal W and clustal X version 2.0. *Bioinformatics* 23 2947–2948. 10.1093/bioinformatics/btm404 17846036

[B40] Levy S. B. Bonnie M. (2004). Antibacterial resistance worldwide: causes, challenges and responses. *Nat. Med.* 10 S122–S129. 15577930 10.1038/nm1145

[B41] Levy S. B. McMurry L. M. Barbosa T. M. Burdett V. Courvalin P. Hillen W. (1999). Nomenclature for new tetracycline resistance determinants. *Antimicrob. Agents Chemother.* 43 1523–1524. 10.1128/aac.43.6.1523 10348788 PMC89314

[B42] Loquasto J. R. Barrangou R. Dudley E. G. Stahl B. Chen C. Roberts R. F. (2013). *Bifidobacterium* animalis subsp. lactis ATCC 27673 Is a genomically unique strain within its conserved subspecies. *Appl. Environ. Microbiol.* 79 6903–6910. 10.1128/aem.01777-13 23995933 PMC3811525

[B43] Lugli G. A. Mancino W. Milani C. Duranti S. Mancabelli L. Napoli S. (2019). Dissecting the evolutionary development of the species bifidobacterium animalis through comparative genomics analyses. *Appl. Environ. Microbiol.* 85 1–16. 10.1128/AEM.02806-18 PMC6585482 30709821

[B44] Lugli G. A. Milani C. Duranti S. Mancabelli L. Mangifesta M. Turroni F. (2018). Tracking the taxonomy of the genus Bifidobacterium based on a phylogenomic approach. *Appl. Environ. Microbiol.* 84 1–14. 10.1128/AEM.02249-17 PMC5795081 29222102

[B45] Luque I. Andújar A. Jia L. Zabulon G. De Marsac N. T. Flores E. (2006). Regulated expression of glutamyl-tRNA synthetase is directed by a mobile genetic element in the cyanobacterium *Tolypothrix* sp. PCC 7601. *Mol. Microbiol.* 60 1276–1288. 10.1111/j.1365-2958.2006.05170.x 16689802

[B46] Mailhe M. Ricaboni D. Vitton V. Cadoret F. Fournier P. E. Raoult D. (2017). ‘Angelakisella massiliensis’ gen. nov., sp. nov., a new bacterial species isolated from human ileum. *New Microbes. New Infect.* 16 51–53. 10.1016/j.nmni.2017.01.003 28203377 PMC5294735

[B47] Martínez J. L. (2008). Antibiotics and antibiotic resistance genes in natural environments. *Science* 321 365–367. 10.1126/science.1159483 18635792

[B48] Milani C. Duranti S. Lugli G. A. Bottacini F. Strati F. Arioli S. (2013). Comparative genomics of *Bifidobacterium* animalis subsp. lactis reveals a strict monophyletic bifidobacterial taxon. *Appl. Environ. Microbiol.* 79 4304–4315. 10.1128/aem.00984-13 23645200 PMC3697524

[B49] Milani C. Lugli G. A. Duranti S. Turroni F. Bottacini F. Mangifesta M. (2014). Genomic encyclopedia of type strains of the genus *Bifidobacterium*. *Appl. Environ. Microbiol.* 80 6290–6302. 10.1128/aem.02308-14 25085493 PMC4178644

[B50] Morita H. Toh H. Oshima K. Nakano A. Arakawa K. Takayama Y. (2015). Complete genome sequence of *Bifidobacterium pseudocatenulatum* JCM 1200T isolated from infant feces. *J. Biotechnol.* 210 68–69. 10.1016/j.jbiotec.2015.06.416 26133926

[B51] Morovic W. Roos P. Zabel B. Hidalgo-cantabrana C. Kiefer A. Barrangou R. (2018). Transcriptional and functional analysis of *Bifidobacterium animalis* subsp. lactis exposure to tetracycline. *Appl. Environ. Microbiol.* 84:e01999-18. 30266728 10.1128/AEM.01999-18 PMC6238047

[B52] Naghizadeh Raeisi S. Ghoddusi H. B. Juncker Boll E. Farahmand N. Stuer-Lauridsen B. Johansen E. (2018). Antimicrobial susceptibility of bifidobacteria from probiotic milk products and determination of the genetic basis of tetracycline resistance in Enterococcus species after in vitro conjugation with *Bifidobacterium animalis* subsp. lactis. *Food Control* 94 205–211. 10.1016/j.foodcont.2018.07.016

[B53] O’Callaghan A. van Sinderen D. (2016). Bifidobacteria and their role as members of the human gut microbiota. *Front. Microbiol.* 7:925. 27379055 10.3389/fmicb.2016.00925 PMC4908950

[B54] Overbeek R. Olson R. Pusch G. D. Olsen G. J. Davis J. J. Disz T. (2014). The seed and the rapid annotation of microbial genomes using subsystems technology (RAST). *Nucleic Acids Res.* 42 206–214. 10.1093/nar/gkt1226 PMC3965101 24293654

[B55] Page A. J. Cummins C. A. Hunt M. Wong V. K. Reuter S. Holden M. T. G. (2015). Roary: rapid large-scale prokaryote pan genome analysis. *Bioinformatics* 31 3691–3693. 10.1093/bioinformatics/btv421 26198102 PMC4817141

[B56] Palmieri C. Princivalli M. S. Brenciani A. Varaldo P. E. Facinelli B. (2011). Different genetic elements carrying the tet(W) gene in two human clinical isolates of Streptococcus suis. *Antimicrob. Agents Chemother.* 55 631–636. 10.1128/aac.00965-10 21115784 PMC3028816

[B57] Peterson E. Kaur P. (2018). Antibiotic resistance mechanisms in bacteria: relationships between resistance determinants of antibiotic producers, environmental bacteria, and clinical pathogens. *Front. Microbiol.* 9:2928. 10.3389/fmicb.2018.02928 30555448 PMC6283892

[B58] Polit A. Yang H. Amund D. (2018). Investigating the transmissibility of tet(W) in bifidobacteria exposed to acid and bile stress. *Biosci. Microbiota Food Heal* 37 39–43. 10.12938/bmfh.17-017 29662736 PMC5897239

[B59] Price M. N. Dehal P. S. Arkin A. P. (2009). Fasttree: computing large minimum evolution trees with profiles instead of a distance matrix. *Mol. Biol. Evol.* 26 1641–1650. 10.1093/molbev/msp077 19377059 PMC2693737

[B60] Price M. N. Dehal P. S. Arkin A. P. (2010). Fast tree 2approximately maximum-likelihood trees for large alignments. *PLoS One* 5:9490. 10.1371/journal.pone.0009490 20224823 PMC2835736

[B61] Rbl S. Jans C. Lacroix C. Follador R. Stevens M. J. A. (2013). Complete genome sequence of the probiotic *Bifidobacterium thermophilum* strain RBL67. *Genome Announc.* 1:e00191-13. 23640377 10.1128/genomeA.00191-13 PMC3642284

[B62] WHO (2011). *World Health Organization. Tackling Antibiotic Resistance from a Food Safety Perspective in Europe.* Copenhagen: Copenhagen World Heal Organ, 1–88.

[B63] Rozman V. Mohar Lorbeg P. Accetto T. Bogovič Matijašć B. (2020). Characterization of antimicrobial resistance in lactobacilli and bifidobacteria used as probiotics or starter cultures based on integration of phenotypic and in silico data. *Int. J. Food Microbiol.* 314:108388. 10.1016/j.ijfoodmicro.2019.108388 31707173

[B64] Sayers E. W. Agarwala R. Bolton E. E. Brister J. R. Canese K. Clark K. (2019). Database resources of the national center for biotechnology information. *Nucleic Acids Res.* 47 D23–D28. 30395293 10.1093/nar/gky1069 PMC6323993

[B65] Schnetz K. Rak B. (1992). IS5: a mobile enhancer of transcription in Eschericia coli. *Proc. Natl. Acad. Sci. U.S.A.* 89 1244–1248. 10.1073/pnas.89.4.1244 1311089 PMC48425

[B66] Schröder J. Maus I. Meyer K. Wördemann S. Blom J. Jaenicke S. (2012). Complete genome sequence, lifestyle, and multi-drug resistance of the human pathogen *Corynebacterium resistens* DSM 45100 isolated from blood samples of a leukemia patient. *BMC Genomics* 13:141. 10.1186/1471-2164-13-141 22524407 PMC3350403

[B67] Scott K. P. Barbosa T. M. Forbes K. J. Flint H. J. (1997). High-frequency transfer of a naturally occurring chromosomal tetracycline resistance element in the ruminal anaerobe Butyrivibrio fibrisolvens. *Appl. Environ. Microbiol.* 63 3405–3411. 10.1128/aem.63.9.3405-3411.1997 9292992 PMC168648

[B68] Seemann T. (2014). Prokka: rapid prokaryotic genome annotation. *Bioinformatics* 30 2068–2069. 10.1093/bioinformatics/btu153 24642063

[B69] Shrivastava S. R. Shrivastava P. S. Ramasamy J. (2018). World health organization releases global priority list of antibiotic-resistant bacteria to guide research, discovery, and development of new antibiotics. *JMS J. Med. Soc.* 32 76–77. 10.4103/jms.jms_25_17 33692645

[B70] Siguier P. Perochon J. Lestrade L. Mahillon J. Chandler M. (2006). ISfinder: the reference centre for bacterial insertion sequences. *Nucleic Acids Res.* 34 D32–D36. 16381877 10.1093/nar/gkj014 PMC1347377

[B71] Spigaglia P. Barbanti F. Mastrantonio P. (2008). Tetracycline resistance gene tet(W) in the pathogenic bacterium Clostridium difficile. *Antimicrob. Agents Chemother.* 52 770–773. 10.1128/aac.00957-07 18070963 PMC2224778

[B72] Stanton T. B. Humphrey S. B. (2003). Isolation of tetracycline-resistant *Megasphaera* elsdenii strains with novel mosaic gene combinations of tet(O) and tet(W) from swine. *Appl. Environ. Microbiol.* 69 3874–3882. 10.1128/aem.69.7.3874-3882.2003 12839756 PMC165211

[B73] Sun Z. Zhang W. Guo C. Yang X. Liu W. Wu Y. (2015). Comparative genomic analysis of 45 type strains of the genus bifidobacterium: a snapshot of its genetic diversity and evolution. *PLoS One* 10:e0117912. 10.1371/journal.pone.0117912 25658111 PMC4319941

[B74] Tamura K. Nei M. (1993). Estimation of the number of nucleotide substitutions in the control region of mitochondrial DNA in humans and chimpanzees. *Mol. Biol. Evol.* 10 512–526. 8336541 10.1093/oxfordjournals.molbev.a040023

[B75] Turroni F. Peano C. Pass D. A. Foroni E. Severgnini M. Claesson M. J. (2012). Diversity of bifidobacteria within the infant gut microbiota. *PLoS One* 7:e36957. 10.1371/journal.pone.0036957 22606315 PMC3350489

[B76] Vandecraen J. Chandler M. Aertsen A. Van Houdt R. (2017). The impact of insertion sequences on bacterial genome plasticity and adaptability. *Crit. Rev. Microbiol.* 43 709–730. 10.1080/1040841x.2017.1303661 28407717

[B77] Wang L. Wang Y. Li Q. Tian K. Xu L. Liu G. (2019). Exopolysaccharide, isolated from a novel strain *Bifidobacterium breve* lw01 possess an anticancer effect on head and neck cancer - genetic and biochemical evidences. *Front. Microbiol.* 10:1044. 10.3389/fmicb.2019.01044 31143171 PMC6520658

[B78] Wang N. Hang X. Zhang M. Liu X. Yang H. (2017). Analysis of newly detected tetracycline resistance genes and their flanking sequences in human intestinal bifidobacteria. *Sci. Rep.* 7 1–10. 28740169 10.1038/s41598-017-06595-0 PMC5524971

[B79] World Health Organisation (WHO). (2017). *Global Action Plan on Antimicrobial Resistance.* Switzerland: WHO, 1–28.

[B80] Zankari E. Hasman H. Cosentino S. Vestergaard M. Rasmussen S. Lund O. (2012). Identification of acquired antimicrobial resistance genes. *J. Antimicrob. Chemother.* 67 2640–2644. 10.1093/jac/dks261 22782487 PMC3468078

