# Supplementary material for: The Tetracycline Resistance Gene, tet(W) in Bifidobacterium animalis subsp. lactis Follows Phylogeny and Differs From tet(W) in Other Species
Source: Front Microbiol. 2021 Jul 15;12:658943. doi: 10.3389/fmicb.2021.658943 (PMC8319848; doi:10.3389/fmicb.2021.658943)
Supplement: Supplementary file 1 [file Data_Sheet_1.PDF]

Supplementary Table S1. Origin of the *Bifidobacterium animalis* subsp. *lactis* and *Bifidobacterium animalis* subsp. *animalis* strains in the study.

**Strain**	**Source**	**Year of isolation**	**Geographic area**	**Subspecies ID**	**Genome size (Mb)**	**GC content (%)**	**Sequencing method**	**Assembly level**	**Number of contigs**	**Coverage**	**Reference**	***tet*(W) gene (bp)**	**IS5 element (bp)**
B. animalis* subsp. *lactis													
**DSM 10140** (TS) (CP001606)	Yoghurt	1997	France	(1)	1.93	60.5	454 pyrosequencing	Complete genome	-	30x	(2, 3)	1920	966
**BM 25** (PHUS01000001)	Dairy product	2016	India	*rpo*A and 16S rRNA	1.91	60.5	IonTorrent	Contig	24	98.96 x	NCBI database, Unpublished article	1920	966
**UBBLa 70** (NZ_RWKO01000001)	Fermented food	2008	India	*rpo*A and 16S rRNA	1.94	60.0	Illumina NextSeq	Contig	71	327.0 x	NCBI database, unpublished article	117	966
**LMG P-17502_1** (NZ_NIGR01000001)	Food samples	2017	Italy	(1)	1.91	60.5	Illumina	Contig	15	391x	(1) NCBI database	1920	1134
**LMG P-17502_2** (NZ_NIGQ01000001)	Food samples	2017	Italy	(1)	1.91	60.5	Illumina	Contig	13	403x	(1) NCBI database	1920	-
**CF3_2** (NZ_QDIV01000012)	Cultured food	2018	USA	*rpo*A and 16S rRNA	1.97	60.6	NextSeq 500	Contig	417	65.9x	(4)	1920	966

**DS23_2** (NZ_QDIO01000003)	Commercial dietary supplements	2017	USA	*rpo*A and 16S rRNA	1.93	60.5	Illumina MiSeq	Contig	114	58.8x	(4)	1920	966
**BB-12** CP001853.2	Food matrices	NA	NA	(1)	1.94	60.5	MiSeq/ONT	Complete genome	-	310x	(5)	1920	966
**ATCC 27673** (CP003941)	Sewage	2015*	Japan	(1)	1.96	60.6	Illumina MiSeq	Scaffold	21	170.0x	(6)	1920	861
**B420** (CP003497)	Human feces	NA	NA	(1)	1.93	60.5	FLX Titanium 454 sequencing	Complete genome	-	18x	(7)	1920	966
**BLC1** (CP003039)	Human feces	NA	NA	(1)	1.93	60.5	FLX Titanium/Illumina	Complete genome	20	30x	(8)	1920	966
**CNCM I-2494** (CP002915)	Human feces	Commercial since 1987	NA	(1)	1.94	60.5	Sanger shotgun sequencing	Complete genome	-	13.6x	(9)	1920	966
**HN019** (CP031154)	Human feces	2018*	USA	(1)	1.93	60.5	Illumina and NanoPore sequencing	Complete genome	-	163.0x	(1) NCBI database	1920	966
**KLDS2.0603** (CP007522)	Human feces	2006	China	(1)	1.94	60.5	454	Complete genome	-	66x	(1) NCBI database	1920	966
**V9** (CP001892)	Human feces	2009	China	(1)	1.94	60.5	Combining 454 sequencin	Complete genome	-	36x/335x	(1, 10)	1920	966

							g and Solexa paired-end sequencing						
**BS01** (NZ_JH472445)	Human feces	2011*	NA	(1)	1.93	60.5	454 GS FLX Titanium	Scaffold	2	39x	(1)	1920	966
**DS27_2** (NZQDIL01000001)	Human feces	2018	USA	(1)	1.92	60.5	Illumina MiSeq	Contig	18	74.3x	(4)	1920	966
**DS24_2** (NZ_QDIN01000001)	Human feces	2018	USA	(1)	1.92	60.5	Illumina MiSeq	Contig	36	60.5x	(4)	1920	966
**DS28_2** (NZ_QDIK01000001)	Human feces	2017	USA	(1)	1.91	60.5	Illumina MiSeq	Contig	17	290.9x	(4)	1920	-
**646** (NZ_MLZL01000008)	Human feces	2015	Italy	(1)	1.92	60.5	Ion Torrent	Contig	21	58.45x	NCBI database	1920	966
**A6**[A] (NZ_CP010433)	Human feces	2006	China	(1)	1.95	60.5	PacBio RS	Complete genome	-	200x	(11)	1920	966
**RH**[A] (NZ_CP007755)	Human feces	2014*	China	(1)	1.93	60.5	Illumina	Complete genome	14	311x	(12)	1920	966
**BL3**[A] (NZ_CP017098)	Human feces	2015	South Korea	(1)	1.94	60.5	PacBio	Complete genome	-	322x	(13)	1920	966
**Bifido_08** (NZ_FTRP01000001)	Human feces	2013-2015	Norway	(1)	1.95	60.4	Whole-genome shotgun sequence	Contig	75	-	NCBI database	1920	966

**Bifido_11** (NZ_FTRE01000001)	Human feces	2013-2015	Norway	(1)	1.93	60.4	Whole-genome shotgun sequence	Contig	46	-	NCBI database	1920	966
**DS1_2** (NZ_QDIU01000001)	Human feces	2016	USA	(1)	1.92	60.5	Illumina MiSeq	Contig	15	28.6x	(4)	1920	966
**DS11_2** (NZ_QDIT01000001)	Human feces	2016	USA	(1)	1.92	60.5	Illumina MiSeq	Contig	15	39.6x	(4)	1920	966
**DS15_2** (NZ_QDIR01000001)	Human feces	2016	USA	(1)	1.91	60.5	Illumina MiSeq	Contig	17	77.6x	(4)	1920	966
**DS2_2** (NZ_QDIP01000001)	Human feces	2016	USA	(1)	1.91	60.5	Illumina MiSeq	Contig	16	209.9x	(4)	1920	966
**S7** (CP022724)	Human feces, adult	2017	South Korea	*rpo*A and 16S rRNA	1.94	60.5	PacBio	Complete genome	-	185.0x	NCBI database	1920	966
**Bl-04** (CP001515)	Fecal sample from healthy adult	NA	NA	(1)	1.93	60.5	454 pyrosequencing	Complete genome	-	15x	(2)	1920	966
**Bl12** (CP004053)	Colonoscopy sample from healthy individual that had not consumed	NA	NA	(1)	1.93	60.5	IonTorrent	Complete genome	-	111.67	(14)	1920	966

	probiotic products											
**CECT 8145** (NZ_CBWX010000073)	Infant feces	2014*	NA	(1)	1.93	60.5	454 pyrosequencing	Scaffold	77	-	(1, 15) NCBI database	1920	966
**AD011** (CP001213)	Infant feces, healthy breast-fed	NA	NA	(1)	1.93	60.5	Sanger pair-ended sequencing of plasmid and fosmid libraries	Complete genome	-	-	(16)	660/1239	966
**BF052** (CP009045)	Feces of breast-fed infant	2011	NA	(1)	1.93	60.5	Illumina	Complete genome	-	37.59x	(1)	1920	966
**IDCC4301** (CP031703)	Infant feces	2018	South Korea	*rpo*A and 16S rRNA	1.94	60.5	PacBio RSII, Illumina HiSeq	Complete genome	-	6.0x	NCBI database	1920	966
**ATCC 27536** (NZ_AWFL01000005)	Chicken feces	2015*	Japan	(1)	1.91	60.5	Illumina MiSeq	Scaffold	18	212.0x	(1) ATCC website	1920	966
**ATCC 27674** (NZ_AWFM01000002)	Rabbit feces	2015*	Japan	(1)	1.91	60.5	Illumina MiSeq	Scaffold	18	308.0x	(1) ATCC website	1920	966
**1395B** (NZ_RSCZ01000003)	*Oryctolagus cumiculus* (Rabbit) feces	2018*	Europe	(1)	1.91	60.5	Illumina Miseq	Contig	12	99x	(1)	1920	966
**2007B** (NZ_RSCQ01000009)	*Canis lupus familiaris*	2018*	NA	(1)	1.97	60.4	Illumina MiSeq	Contig	25	194x	(1)	-	-

	(Pomeranian) feces												
**2010B** (NZ_RSCP01000005)	*Canis lupus familiaris* (Alaskan malamute) feces	2018*	NA	(1)	1.98	60.3	Illumina MiSeq	Contig	29	121x	(1)	-	-
**2011B** (NZ_RSCO01000028)	*Canis lupus familiaris* (Flat coated retriever) feces	2018*	NA	(1)	2.08	60.4	Illumina MiSeq	Contig	44	181x	(1)	-	-
**1528B** (NZ_RSCY01000002)	*Sus scrofa domesticus* feces	2018*	NA	(1)	1.95	60.5	Illumina MiSeq	Contig	12	97x	(1)	1920	282
**1821B** (NZ_RSCT01000003)	*Pan troglodytes* feces (Common chimpanzee)	2018*	NA	(1)	2.00	60.5	Illumina MiSeq	Contig	38	278x	(1)	1920	966
**1843B** (NZ-_RSCS01000003)	*Pan troglodytes* feces (Common chimpanzee)	2018*	NA	(1)	1.91	60.5	Illumina MiSeq	Contig	14	85x	(1)	1920	966
**1869B** (NZ_RSCR01000005)	*Pan troglodytes* feces (Common	2018*	NA	(1)	1.91	60.5	Illumina MiSeq	Contig	14	229x	(1)	1920	966

	chimpanzee)											
**1811B** (NZ_RSCV01000003)	*Chlorocebus pygerytrhus* feces (Vervet monkey)	2018*	NA	(1)	1.92	60.5	Illumina MiSeq	Contig	16	156x	(1)	1920	966
**1808B** (NZ_RSCW01000006)	*Chlorocebus pygerytrhus* feces (Vervet monkey)	2018*	NA	(1)	1.91	60.5	Illumina MiSeq	Contig	15	87x	(1)	1920	966
**1802B** (NZ_RSCX01000005)	*Macaca sylvanus* feces (Barbary macaque)	2018*	NA	(1)	1.91	60.5	Illumina MiSeq	Contig	15	132x	(1)	1920	966
**Bi-07** (CP003498)	From the Danisco Global Culture Collection	NA	NA	(1)	1.93	60.5	FLX Titanium 454 sequencing	Complete genome	-	18x	(7)	1920	966
B. animalis* subsp. *animalis												
**ATCC 25527/LMG 10508** (TS) (CP002567)	-	2012*	NA	(1)	1.93	60.5	454 pyrosequencing on a GS-FLX sequencer	Complete genome		-	(17)	-	-
**CNCM I-4602** (NZ_CP028460)	Homo sapiens	2014	France	*rpo*A and	1.93	60.5		Complete genome		400.0 x	(18)	-	-

**YL2** (NZ_CP015407)	*Mus musculus*	2015	Switzerland	16S rRNA (1)	2.03	60.2	PacBio	Complete genome	1	150.0x	(19)	-	-
**IM386** (SAMEA3158463)	Human feces	2015*	NA	(1)	1.93	60.4		Contig	8	383x	(1)	-	-
**ATCC 27672** (SAMN03978812)	Rat feces	2015*	Japan	(1)	1.99	60.1	Illumina MiSeq	Scaffold	16	147.0x	(1)	-	-
**MCC 1489** (SAMN03978810)	Guinea pig feces	2015*	Japan	(1)	1.91	60.5	Illumina MiSeq	Scaffold	17	219.0x	(1)	-	-
**2006B** (SAMN10459547)	*Canis lupus familiaris* (German shepherd) feces	2018*	NA	(1)	2.16	60.3	Illumina MiSeq	Contig	47	195x	(1)	-	-
**2022B** (SAMN10459546)	*Castor fiber* feces	2018*	NA	(1)	2.41	60.1	Illumina MiSeq	Contig	17	142x	(1)	-	-

NA: Not available, *Year of submission to NCBI, A: Strain A6, RH and BL3 has recently been subspecies classified as *B. animalis* subsp. *lactis* (1). TS: type strain.

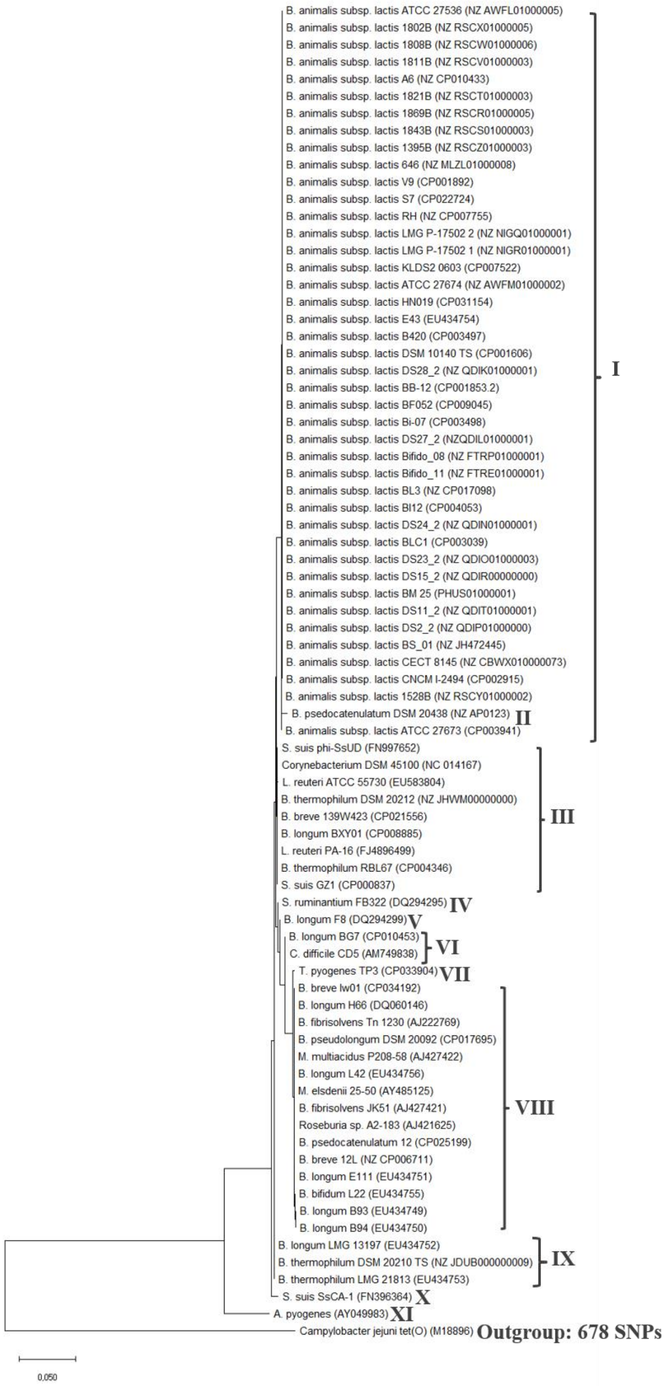

Supplementary Figure S1. Phylogenetic tree of the *tet*(W) gene. The tree was built by evolutionary analysis by Maximum Likelihood method and Tamura-Nei model by MEGA X (20, 21). The branch lengths are measured in the number of substitutions per site. Strain name and genome or *tet*(W) gene accession number is provided for the sequences. Type strains (TS) are included for the species, when the type strain encodes *tet*(W). Clades are defined by the number of SNPs, which can be seen in Table 2. The phylogenetic trees were rooted with the ribosomal protection gene *tet*(O) from *Campylobacter jejuni* (M18896) as an outgroup and similar results was obtained with the *Streptococcal* ribosomal protection gene *tet*(M) (X04388) (data not shown).

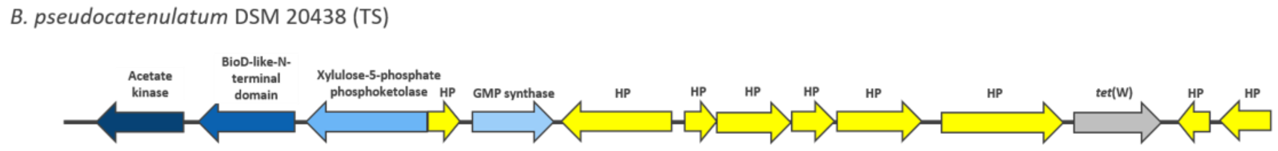

Supplementary Figure S2. The chromosomal region downstream of *tet*(W) in the *Bifidobacterium pseudocatenulatum* type strain DSM 20438.

1. Lugli GA, Mancino W, Milani C, Duranti S, Mancabelli L, Napoli S, Mangifesta M, Viappiani A, Anzalone R, Longhi G, van Sinderen D, Ventura M, Turroni F. 2019. Dissecting the evolutionary development of the species bifidobacterium animalis through comparative genomics analyses. Appl Environ Microbiol 85:1–16.
2. Barrangou R, Briczinski EP, Traeger LL, Loquasto JR, Richards M, Horvath P, Coûté-Monvoisin AC, Leyer G, Rendulic S, Steele JL, Broadbent JR, Oberg T, Dudley EG, Schuster S, Romero DA, Roberts RF. 2009. Comparison of the complete genome sequences of Bifidobacterium animalis subsp. lactis DSM 10140 and Bl-04. J Bacteriol 191:4144–4151.
3. Meile L, Ludwig W, Rueger U, Gut C, Kaufmann P, Dasen G, Wenger S, Teuber M. 1997. Bifidobacterium lactis sp. nov., a moderately oxygen tolerant species isolated from fermented milk. Syst Appl Microbiol 20:57–64.
4. Barnaba TJ, Gangiredla J, Mammel MK, Lacher DW, Elkins CA, Lampel KA, Whitehouse CA, Tartera C. 2018. Draft genome sequences of Bifidobacterium strains isolated from dietary supplements and cultured food products. Genome Announc 6:10–11.
5. Garrigues C, Johansen E, Pedersen MB. 2010. Complete genome sequence of Bifidobacterium animalis subsp. lactis BB-12, a widely consumed probiotic strain. J Bacteriol 192:2467–2468.
6. Loquasto JR, Barrangou R, Dudley EG, Stahl B, Chen C, Roberts RF. 2013. Bifidobacterium animalis subsp. lactis ATCC 27673 Is a genomically unique strain within its conserved subspecies. Appl Environ Microbiol 79:6903–6910.
7. Stahl B, Barrangou R. 2012. Complete genome sequences of probiotic strains Bifidobacterium animalis subsp. lactis B420 and Bi-07. J Bacteriol 194:4131–4132.
8. Bottacini F, Dal Bello F, Turroni F, Milani C, Duranti S, Foroni E, Viappiani A, Strati F, Mora D, van Sinderen D, Ventura M. 2011. Complete Genome Sequence of Bifidobacterium animalis subsp. Lactis BLC1. J Bacteriol 193:6387–6388.
9. Chervaux C, Grimaldi C, Bolotin A, Quinquis B, Legrain-Raspaud S, van Hylckama Vlieg JET, Denariaz G, Smokvina T. 2011. Genome sequence of the probiotic strain Bifidobacterium animalis subsp. lactis CNCM I-2494. J Bacteriol 193:5560–5561.
10. Sun Z, Chen X, Wang J, Gao P, Zhou Z, Ren Y, Sun T, Wang L, Meng H, Chen W, Zhang H. 2010. Complete genome sequence of probiotic Bifidobacterium animalis subsp. lactis strain V9. J Bacteriol 192:4080–4081.
11. Sun E, Zhao L, Ren F, Liu S, Zhang M, Guo H. 2015. Complete genome sequence of Bifidobacterium animalis subsp. lactis A6, a probiotic strain with high acid resistance ability. J Biotechnol 200:8–9.
12. Liu L, Qin Y, Wang Y, Li H, Shang N, Li P. 2014. Complete genome sequence of Bifidobacterium animalis RH, a probiotic bacterium producing exopolysaccharides. J Biotechnol 189:86–87.
13. Kang J, Chung WH, Lim TJ, Lim S, Nam YD. 2017. Complete genome sequence of the Bifidobacterium animalis subspecies lactis BL3, preventive probiotics for acute colitis and

colon cancer. New Microbes New Infect 19:34–37.
14. Milani C, Duranti S, Lugli GA, Bottacini F, Strati F, Arioli S, Foroni E, Turroni F, van Sinderen D, Ventura M. 2013. Comparative genomics of Bifidobacterium animalis subsp. lactis reveals a strict monophyletic bifidobacterial taxon. Appl Environ Microbiol 79:4304–4315.
15. Chenoll E, Silva A, Martorell P. 2014. Strain CECT 8145 , Able To Improve Metabolic Syndrome In Vivo 2:11071–11079.
16. Kim JF, Jeong H, Yu DS, Choi SH, Hur CG, Park MS, Yoon SH, Kim DW, Ji GE, Park HS, Oh TK. 2009. Genome sequence of the probiotic bacterium bifidobacterium animalis subsp. lactis AD011. J Bacteriol 191:678–679.
17. Loquasto JR, Barrangou R, Dudley EG, Roberts RF. 2011. Short communication: The complete genome sequence of Bifidobacterium animalis subspecies animalis ATCC 25527 T and comparative analysis of growth in milk with B. animalis subspecies lactis DSM 10140 T. J Dairy Sci 94:5864–5870.
18. Egan M, Bottacini F, O'Connell Motherway M, Casey PG, Morrissey R, Melgar S, Faurie JM, Chervaux C, Smokvina T, van Sinderen D. 2018. Staying alive: growth and survival of Bifidobacterium animalis subsp. animalis under in vitro and in vivo conditions. Appl Microbiol Biotechnol 102:10645–10663.
19. Li H, Limenitakis JP, Fuhrer T, Geuking MB, Lawson MA, Wyss M, Brugiroux S, Keller I, Macpherson JA, Rupp S, Stolp B, Stein J V., Stecher B, Sauer U, McCoy KD, Macpherson AJ. 2015. The outer mucus layer hosts a distinct intestinal microbial niche. Nat Commun 6.
20. Kumar S, Stecher G, Li M, Knyaz C, Tamura K. 2018. MEGA X: Molecular evolutionary genetics analysis across computing platforms. Mol Biol Evol 35:1547–1549.
21. Tamura K, Nei M. 1993. Estimation of the number of nucleotide substitutions in the control region of mitochondrial DNA in humans and chimpanzees. Mol Biol Evol 10.
